# A practical guide for planning pelvic bone percutaneous interventions (biopsy, tumour ablation and cementoplasty)

**DOI:** 10.1007/s13244-018-0600-y

**Published:** 2018-03-21

**Authors:** Marta Oñate Miranda, Thomas P. Moser

**Affiliations:** Department of Radiology, Centre Hospitalier de l’Université de Montréal (CHUM), 1000 rue St-Denis, Montréal, QC H2X 0C1 Canada

**Keywords:** Interventional musculoskeletal radiology, Pelvic bone, Biopsy, Cementoplasty, Tumour ablation

## Abstract

**Abstract:**

Percutaneous approaches for pelvic bone procedures (bone biopsies, tumour ablation and cementoplasty) are multiple and less well systematised than for the spine or extremities. Among the different imaging techniques that can be used for guidance, computed tomography (CT) scan is the modality of choice because of the complex pelvic anatomy. In specific cases, such as cementoplasty where real-time evaluation is a determinant, a combination of CT and fluoroscopy is highly recommended. The objective of this article is to propose a systematic approach for image-guided pelvic bone procedures, as well as to provide some technical tips. We illustrate the article with multiple examples, and diagrams of the approaches and important structures to avoid to perform these procedures safely.

**Teaching Points:**

• *Pelvic bone procedures are safe to perform if anatomical landmarks are recognised.*

• *The safest approach varies depending on the pelvic level.*

• *CT is the modality of choice for guiding pelvic percutaneous procedures.*

• *Fluoroscopy is recommended when real-time monitoring is mandatory.*

• *MRI can also be used for guiding pelvic percutaneous procedures.*

## Introduction

Percutaneous approaches for the spine [1] or extremities [2] have been well described in the literature. On the other hand, approaches to the pelvic bone are more complex and poorly systematised. There are several percutaneous procedures aimed at pelvic bone: biopsies of primary bone tumours and metastases [3, 4], percutaneous tumour ablation [5–7], cementoplasty [8–13] and percutaneous screw fixation [14, 15]. All these techniques can be used alone or in combination [16] using the same approaches. The objective of this article is to propose a systematic approach to perform image-guided pelvic bone procedures in the safest way and discuss their technical aspects.

## Guidance modalities

The bony pelvis is a ring formed by the sacrum and the innominate bones joined by the pubic symphysis and the sacroiliac joints. It contains several visceral structures of the genitourinary and lower digestive systems and many vessels and nerves transiting between the pelvis and the lower limbs. Different modalities can be used for guiding pelvic bone percutaneous interventions.

### Computed tomography (CT) scanner

CT has been found to provide the most convenient and safest guidance modality [17]. Gantry angulation can be used to facilitate needle placement. Unless using the highly radiating CT fluoroscopy, its main drawback is the absence of real time imaging and control in the *Z*-axis, which are important in some procedures, such as cementoplasty. This disadvantage can be solved by combining the CT scanner with a C-arm fluoroscopy [10, 12, 18]. In this setting, CT would be typically used for guiding needle placement, while the fluoroscopy allows real-time evaluation of cement distribution during the injection. CT scanner can also be used to guide biopsies by identifying anatomical landmarks when lesions are only visible with other imaging techniques such as magnetic resonance imaging (MRI) or positron emission tomography (PET) scan [16, 19].

### Fluoroscopy

**Fig. 1:**
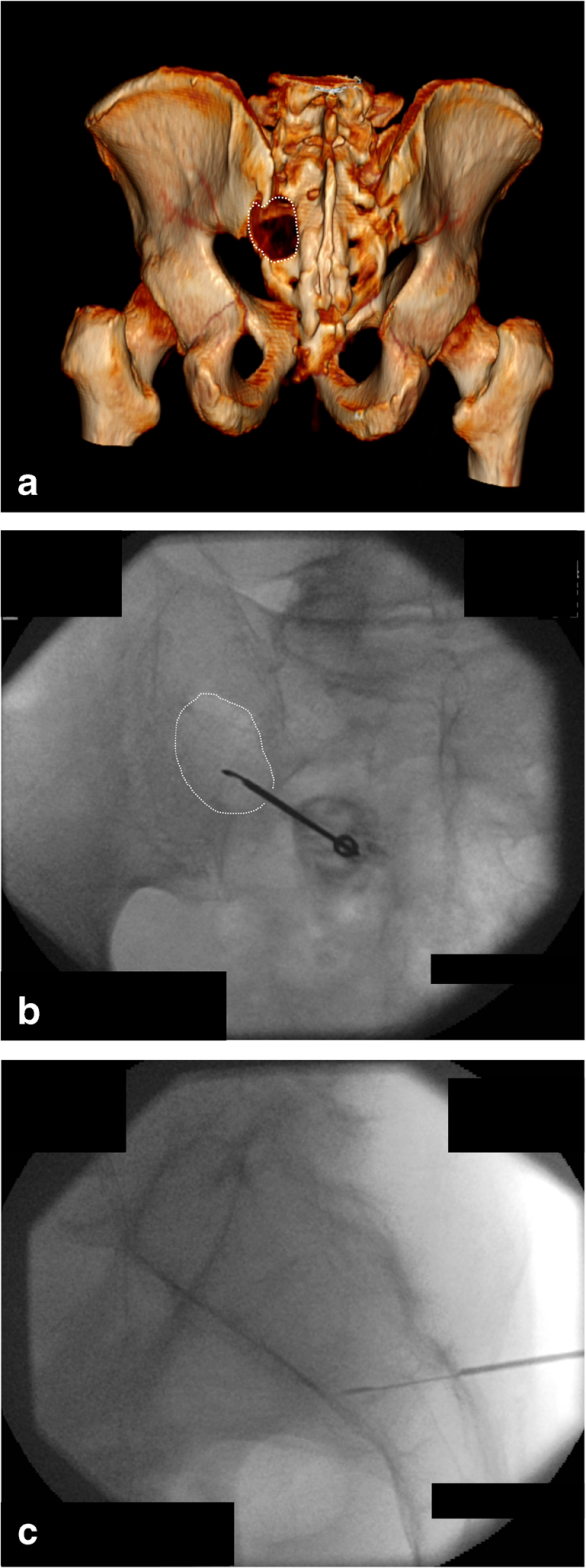
A 77-year-old man with clinical background of melanoma presenting with a new lytic lesion in the left sacral wing. **a** CT 3D reconstruction shows the lytic lesion (*white dotted line*). **b**, **c** PA and lateral fluoroscopic views were used to guide the biopsy, which was performed with a coaxial 16-G cutting needle and confirmed melanoma metastasis

The complex anatomy of the pelvis is rather difficult to appreciate with fluoroscopy. In some cases, bi-plane fluoroscopy provides enough guiding information as it allows multiplane imaging [20] (Fig. 1). The development of flat panel detectors and cone beam CT also allows real-time imaging and capability for three-dimensional reformations with lower radiation doses than CT [20–22]. Although the image quality of the multiplane reformations is inferior to a conventional CT scanner, it is usually appropriate for guiding bone procedures safely [21, 23].

### MRI

MRI guidance could be especially valuable when tumour lesions are not seen with other modalities [16]. The absence of ionising radiation makes it an option for procedures in particular situations such as pregnancy. Because of its high soft tissue contrast, it can show chemical and thermal variations during tumour ablation procedures [5, 20]. However, its main disadvantages remain its higher cost, longer procedure times and limited availability. In addition, the material used during MR-guided interventions should be ideally compatible, which makes it more expensive, or used with special precautions [5, 20, 24].

### Ultrasound

**Fig. 2:**
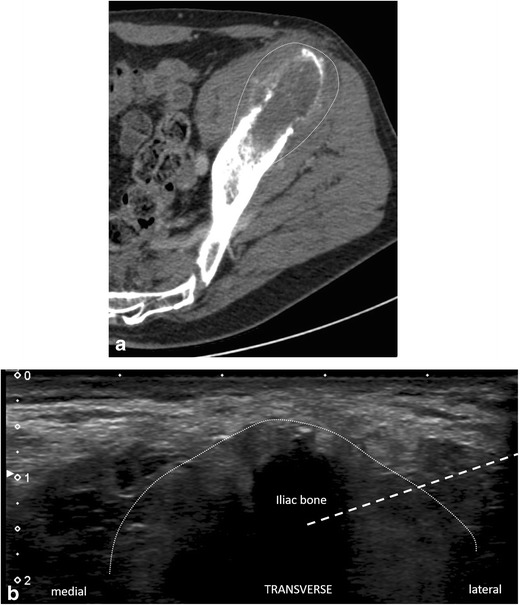
A 75-year-old man with clinical background of rectum adenocarcinoma presenting with a new lytic lesion in the iliac bone. **a** CT scan and **b** ultrasound show the osteolytic lesion with soft tissue extension (*dotted line*). An ultrasound-guided biopsy was made with a coaxial 14-G cutting needle (chosen path marked with a *dashed line*) and confirmed adenocarcinoma metastasis

On occasions, ultrasound can be used to guide pelvic bone biopsies, particularly in the presence of tumour extension to the soft-tissues (Fig. 2) [16]. Among its advantages are its availability, low cost, absence of ionising radiation, and real-time and multiplane imaging [16, 20, 24].

### Navigation systems

Electromagnetic navigation allows real-time device tracking [22]. Needle position information in the magnetic field is processed and placed on a preprocedural imaging (CT or MRI), which is used as a map. Generally, fluoroscopy or CT scan images are acquired to confirm the final needle placement in the target, as the main pitfall is the potential mismatch with the preprocedural images [22].

Other techniques, such as laser guidance, can facilitate needle placement [21, 22]. After CT or cone beam CT images are obtained, the target point is defined and a straight path from the skin is selected [21, 22]. A laser beam indicates the chosen entry site on the skin and the needle orientation [21, 22].

## Approaches and essential landmarks

The safest approach for a percutaneous pelvic bone procedure varies depending on the level, as different important-to-avoid structures exit the pelvis through diverse foramina and vary their relative position. The following descriptions of safe approaches are based on CT scan, as it is the recommended modality for guiding percutaneous pelvic bone procedures [17]. Four main levels can be considered:

### Iliac wings level

**Fig. 3:**
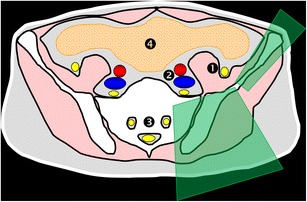
Diagram of the pelvis at the iliac wings level showing the principal structures to avoid (*1* femoral nerve, *2* iliac vessels and lumbosacral trunk, *3* sacral canal and foramina, *4* visceral structures) and the safe approaches (*green areas*)

**Fig. 4:**
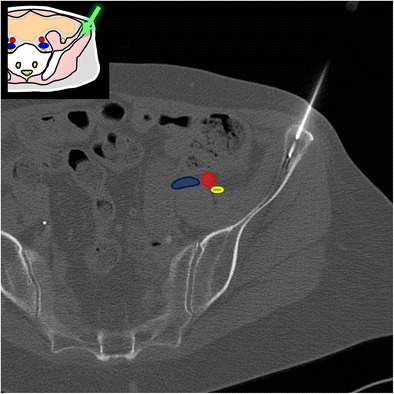
A 69-year-old woman with clinical background of melanoma presenting with a new lytic lesion in the iliac bone. An anterolateral approach through the anterior-superior iliac spine was used to perform a biopsy using a coaxial 16-G cutting needle confirming melanoma metastasis

**Fig. 5:**
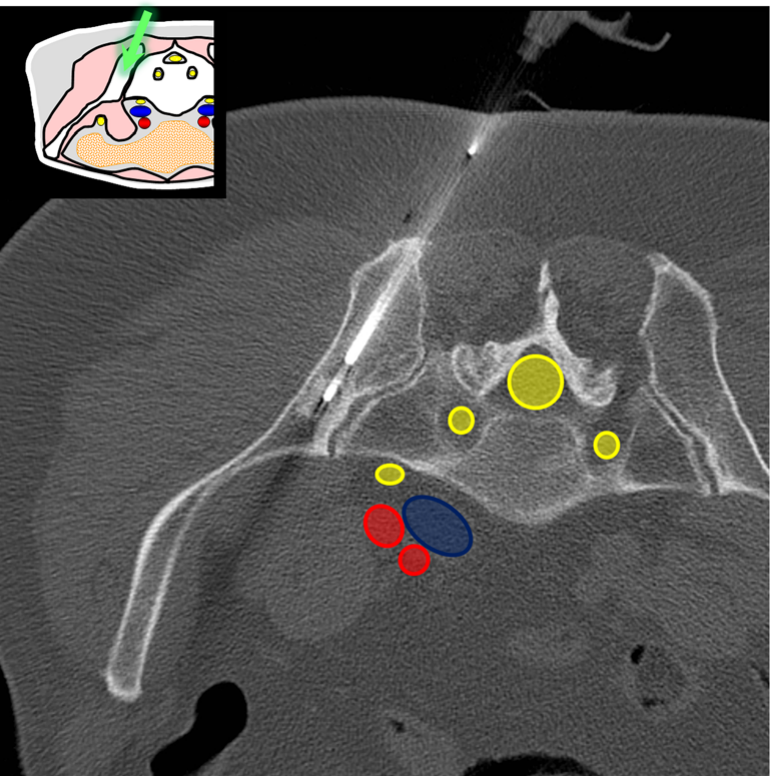
A 72-year-old man with clinical background of prostate cancer presenting with a new blastic lesion in the iliac bone. A posterior approach through the posterior iliac tuberosity was used to perform a biopsy with an 11-G bone biopsy needle confirming prostate metastasis. The choice of a needle path along the greater axis of the iliac bone allows taking multiple samples

**Fig. 6:**
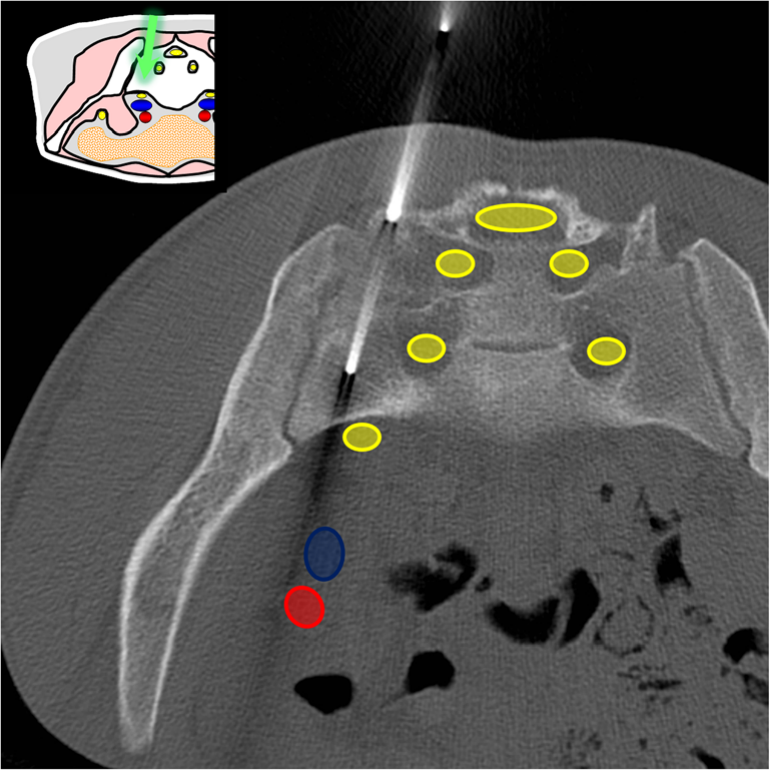
A 24-year-old man presenting with multiple lytic lesions and no known primary tumour. A posterior approach targeting a sacral lytic lesion was chosen and the biopsy with an 11-G bone biopsy needle demonstrated non-Hodgkin’s lymphoma

**Fig. 7:**
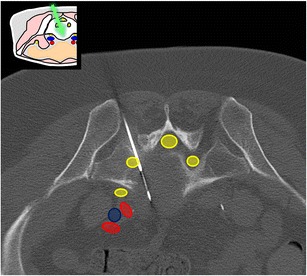
A 64-year-old woman presenting with a sacral lytic lesion extending into the first and second sacral foramina. A posterolateral approach through the S1 foramen was chosen to allow the use of a coaxial 16-G cutting needle. Biopsy proved it to be a poorly differentiated adenocarcinoma of upper gastrointestinal tract origin

**Fig. 8:**
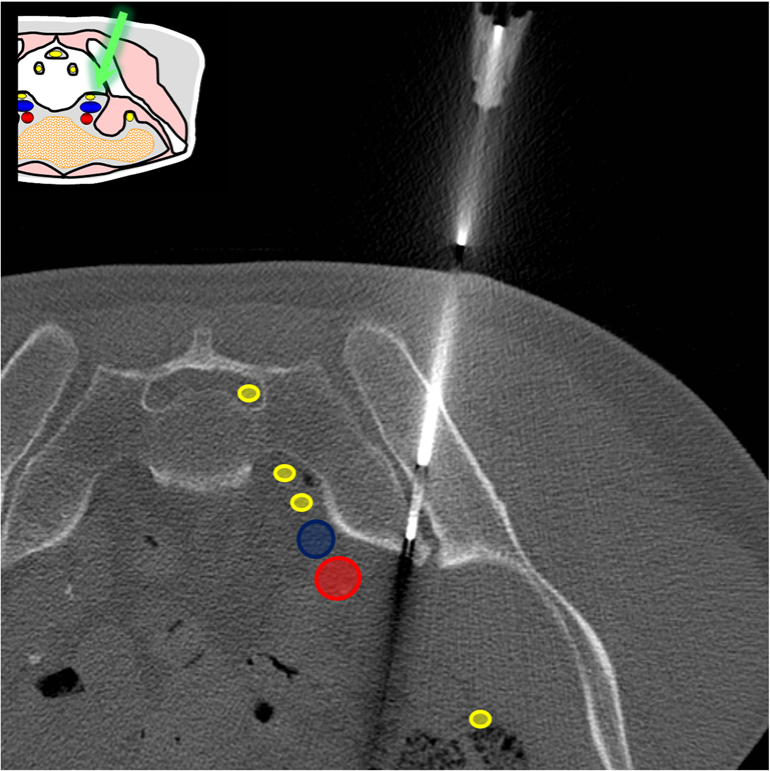
A 59-year-old man presenting with unilateral sacroiliitis, negative blood cultures and negative sacroiliac joint fluid aspiration. Axial CT shows the 11-G bone biopsy needle through the sacroiliac joint. This approach was planned to allow sampling of both the joint space and subchondral bone “sandwich technique” and increase biopsy yield

**Fig. 9:**
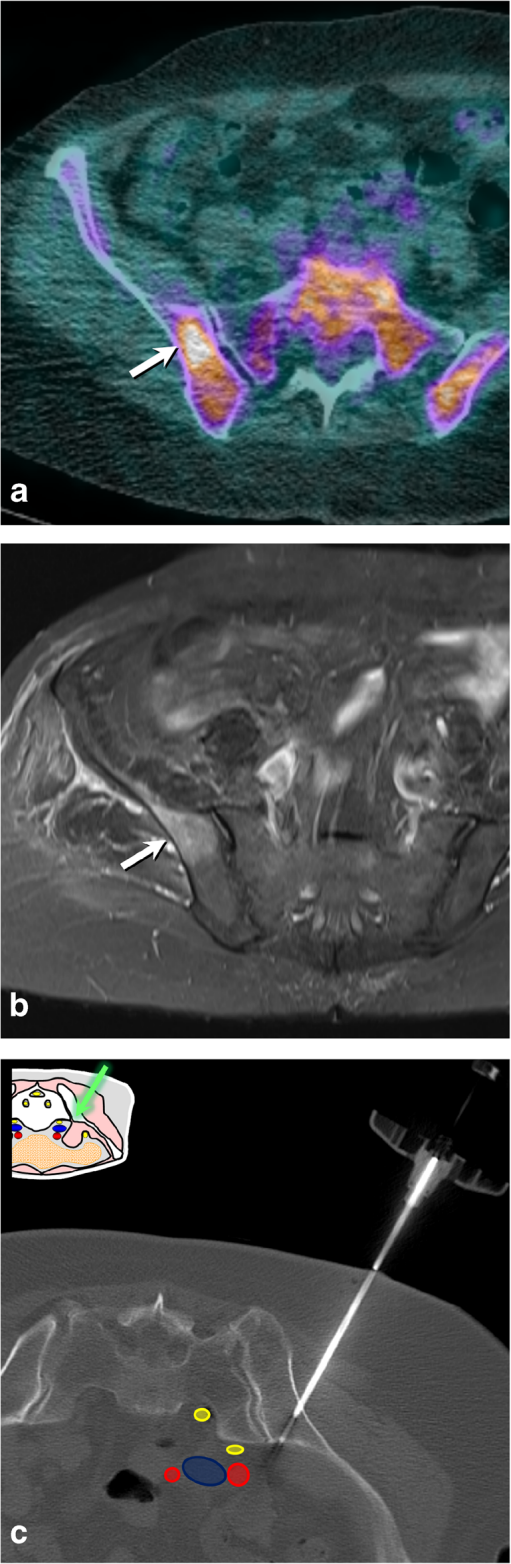
A 77-year-old woman presenting with multiple foci of increased bone metabolism on PET scan and no known primary tumour (**a**, *arrow*) and abnormal bone marrow signal on MRI (**b**, *arrow*). The lesions were not visible on CT and a biopsy of the most conspicuous lesion was carefully planned using anatomical landmarks (**c**). The lesion was targeted through a direct approach and proved to be non-Hodgkin’s lymphoma

At this level, the bone landmarks are the iliac wings with the superior anterior iliac spine and the posterior iliac tuberosity, the sacrum and the sacroiliac joints. The important structures to notice and avoid when performing a bone procedure are the iliac vessels and femoral nerve, the lumbosacral trunk, the sacral nerves and all the pelvic visceral structures (Fig. 3). Five different approaches are routinely performed:

- In the supine position, an anterolateral approach through the anterior superior iliac spine to a target in the iliac wing (Fig. 4)
- In the prone position, a posterior approach through the iliac tuberosity to a target in the iliac bone (Fig. 5)
- In the prone position, a posterior approach through the sacrum to a target in the sacral wing or body (Figs. 6 and 7)
- In the prone position, a posterolateral approach through the sacroiliac joint (trans-sacroiliac) to a target in the sacral body or to biopsy the sacroiliac joint (Fig. 8)
- In the prone position, a direct posterior or posterolateral approach (Fig. 9)

### Acetabular roof level

**Fig. 10:**
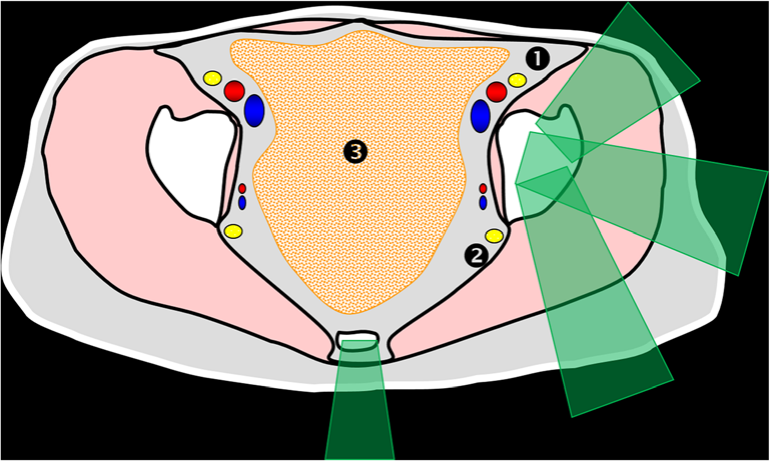
Diagram of the pelvis at the acetabular roof level showing the principal structures to avoid (*1* femoral nerve and external iliac vessels, *2* sciatic nerve, *3* visceral structures) and the safe approaches (*green areas*)

At this level, the bone landmarks are the acetabular roof, sacrum and coccyx. Attention should be paid to the pelvic visceral structures, the femoral nerve and external iliac vessels anteriorly, and the internal iliac vessels and gluteal branches, sacral plexus and structures exiting the pelvis through the greater sciatic notch posteriorly (Fig. 10).

**Fig. 11:**
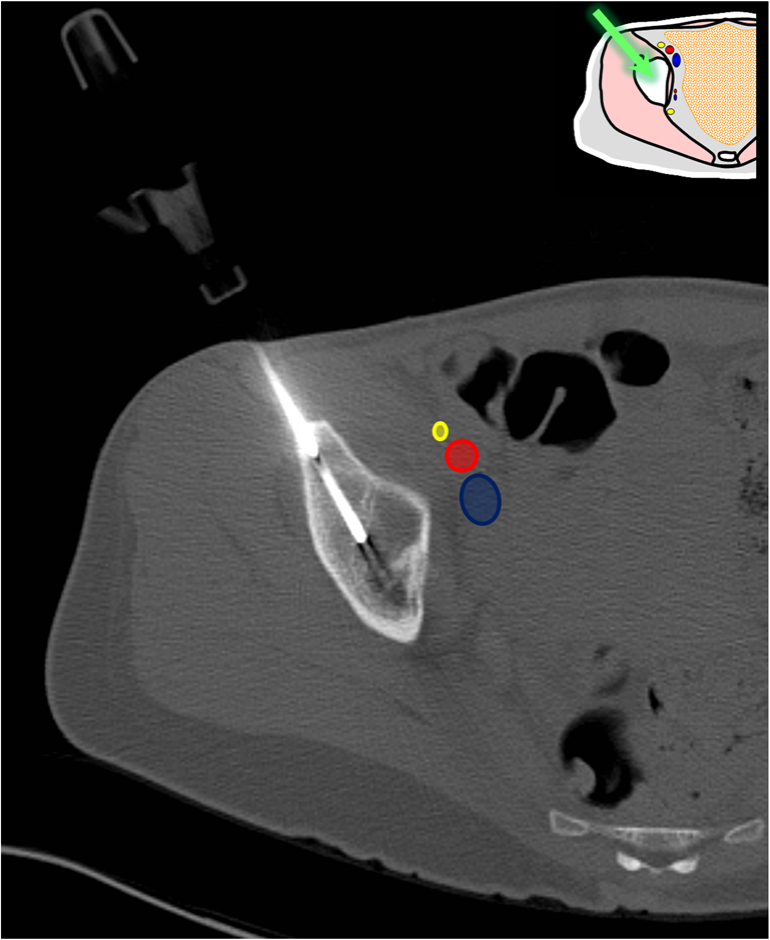
A 49-year-old woman treated for lung cancer presenting with several new lytic bone lesions and right hip pain. An anterolateral approach through the anterior iliac border was used and the biopsy with an 11-G bone biopsy needle confirmed lung metastasis. During the same procedure, a cementoplasty was performed to relieve the pain and reduce the risk of fracture (not shown)

**Fig. 12:**
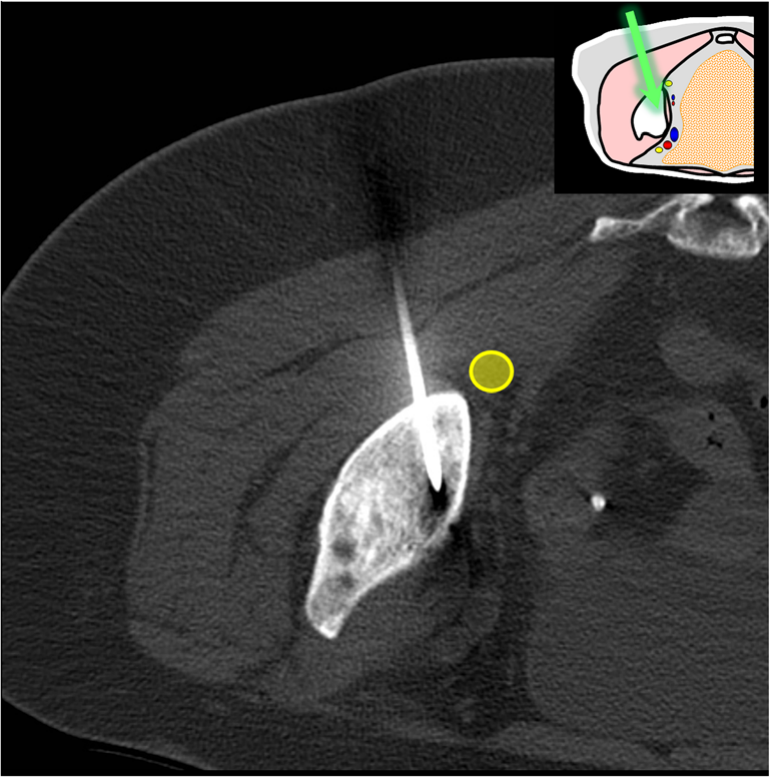
A 70-year-old woman followed-up for a breast carcinoma presenting with new blastic lesions. A posterior approach to the acetabular roof was used. The biopsy with an 11-G bone biopsy needle confirmed breast carcinoma metastasis. A cementoplasty was performed at the same time through the coaxial needle to relieve the pain (not shown)

- If the femoral head is not visible, anterolateral, lateral or posterolateral approaches are safe (Figs. 11 and 12).

We recommend to avoid the anterior approach as the femoral nerve and vessels are lying just anterior and the risk of traversing the peritoneum exists. A posterior approach must be planned very carefully because many vessels and nerves transit through the greater sciatic foramen.

**Fig. 13:**
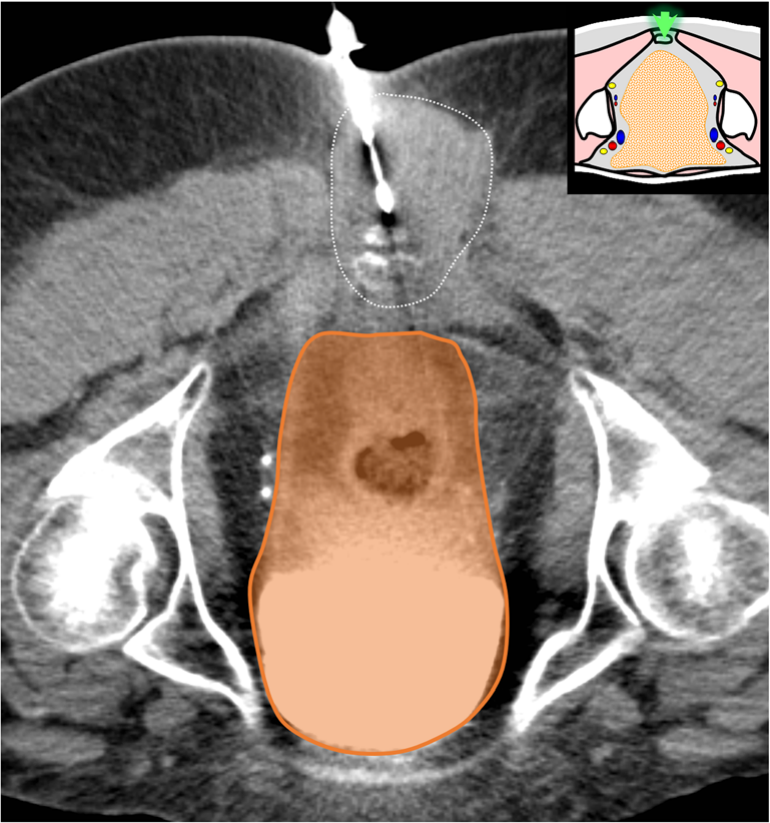
A 62-year-old woman presenting with an expansive sacral lesion (*dotted line*) and no known primary tumour. After discussion with the orthopaedic surgeon, a posterior approach was selected for the biopsy with a 16-G cutting needle and the lesion proved to be a chordoma

- For coccygeal biopsies, a posterior approach is safe, paying attention to visceral structures lying anterior to the coccyx (Fig. 13).

### Hip joint level

**Fig. 14:**
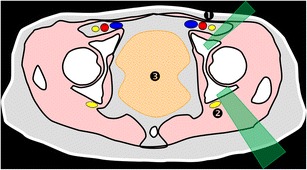
Diagrammatic representation of the pelvis at the hip joint level showing the principal structures to avoid (*1* femoral vessels and nerve, *2* sciatic nerve, *3* visceral structures) and the safe approaches (*green areas*)

At this level, the bony landmarks are the anterior and posterior walls of the acetabulum and the femoral head. Special attention must be paid to the femoral nerve and vessels anteriorly and to the sciatic nerve posteriorly (Fig. 14).

**Fig. 15:**
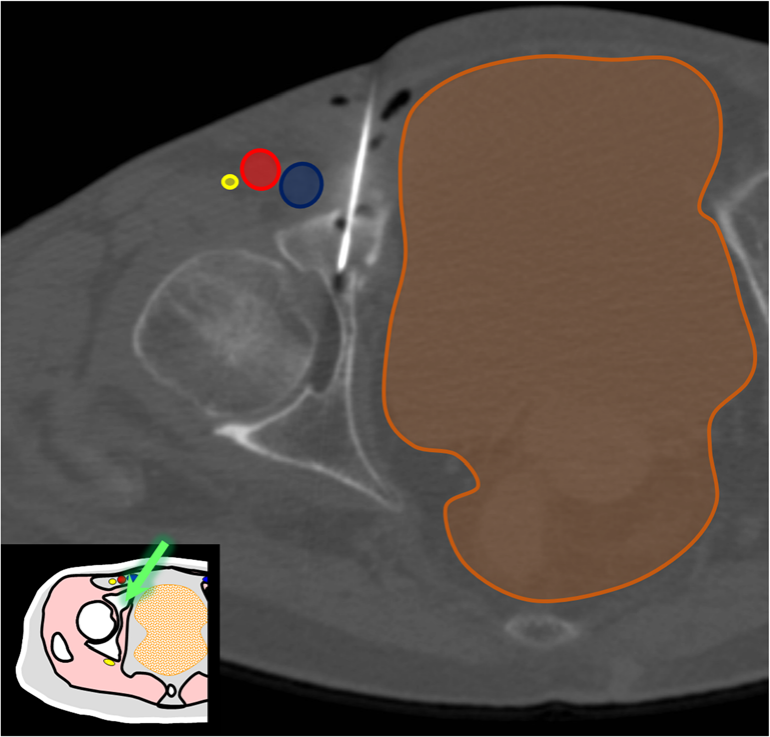
A 69-year-old woman with clinical background of lung cancer presenting with painful pathological fracture of the superior pubic ramus. An anterior approach medial to the femoral vessels was used to perform cementoplasty through an 18-G spinal needle

**Fig. 16:**
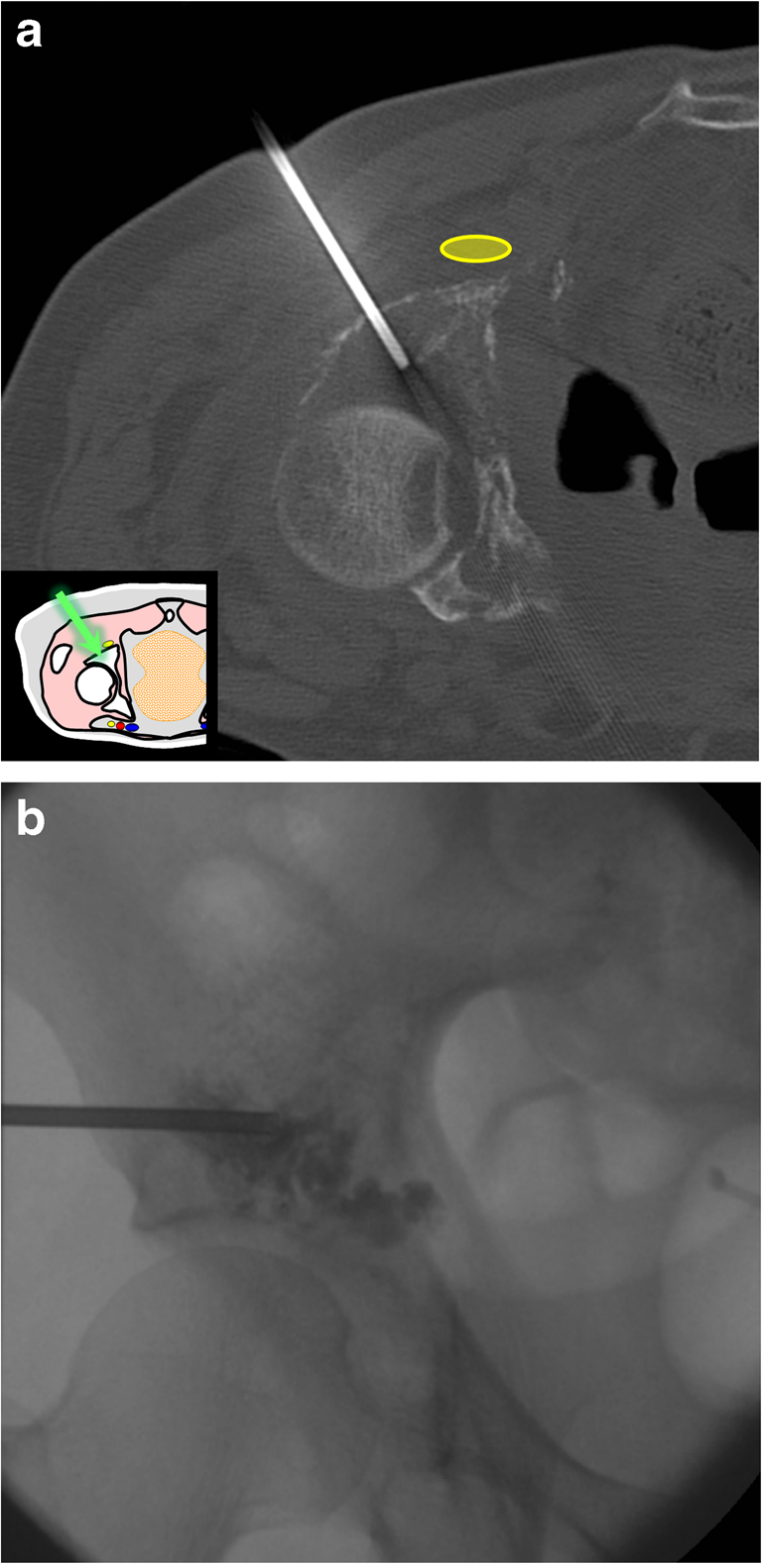
A 70-year-old man treated for metastatic bladder carcinoma and presenting with severe hip pain. **a** A posterior approach for acetabular cementoplasty with an 11-G bone needle was used for pain control and stability. **b** PA fluoroscopic view is used during injection to control the real-time cement distribution in the *Z*-axis. In this specific location, particular attention should be paid for cement leakage into the joint space

**Fig. 17:**
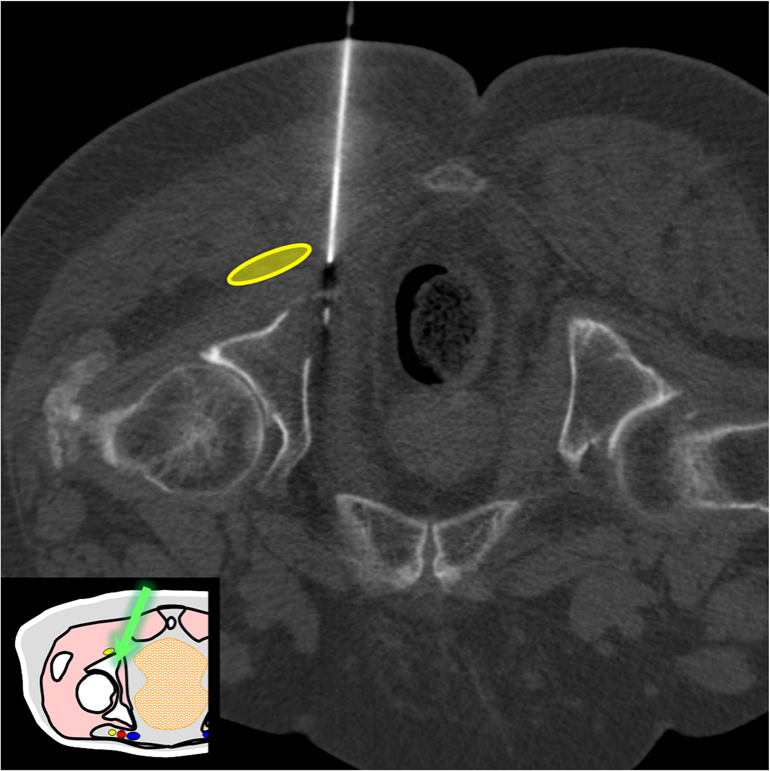
An 80-year-old woman with clinical background of clear cell renal carcinoma presenting with a new lytic lesion at the ischial spine. A posterior approach, medial to the sciatic nerve, was used to perform biopsy with a coaxial 16-G cutting needle, which confirmed clear cell renal carcinoma metastasis

- Anterior (Fig. 15) and posterior (Figs. 16 and 17) approaches must be carefully planned. Important anterior and posterior structures could be avoided laterally or medially depending on the level.

### Ischial tuberosity and pubic symphysis level

**Fig. 18:**
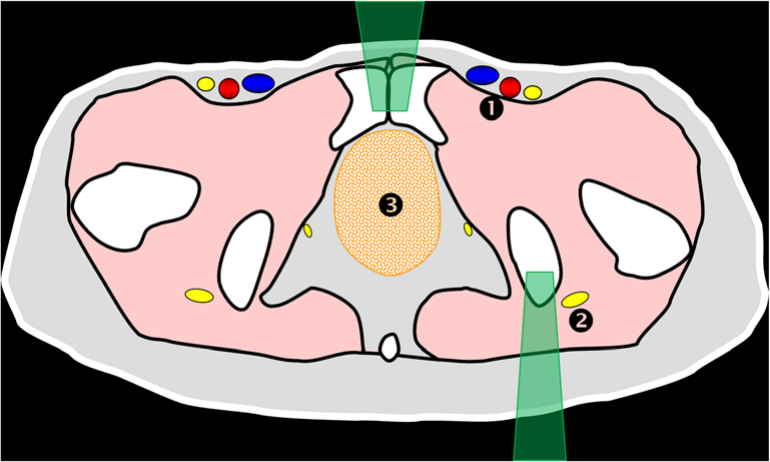
Diagrammatic representation of the pelvis at the pubis symphysis level showing the principal structures to avoid (*1* femoral vessels and nerve, *2* sciatic nerve, *3* visceral structures) and the safe approaches (*green areas*)

At this level, the bony landmarks are the ischial tuberosities, pubic symphysis and femoral neck. Femoral nerves and vessels anteriorly and sciatic nerve posteriorly, between the ischial tuberosity and the femur, should be avoided (Fig. 18).

**Fig. 19:**
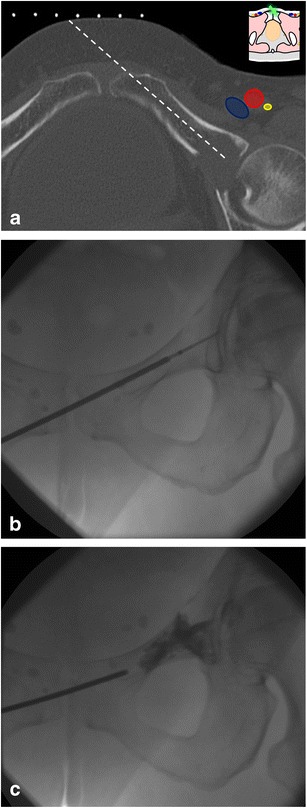
A 66-year-old woman presenting with a painful superior pubic ramus lung carcinoma metastasis. A thermal ablation and cementoplasty was performed for pain control and bone support. **a** An approach through the pubic symphysis and superior pubic ramus (*dashed line*) was chosen because the femoral vessels and nerve prevented direct access. **b** AP fluoroscopy image shows the radiofrequency ablation electrode inserted coaxially within the lesion. **c** AP fluoroscopy image shows the cementoplasty performed through the coaxial needle

**Fig. 20:**
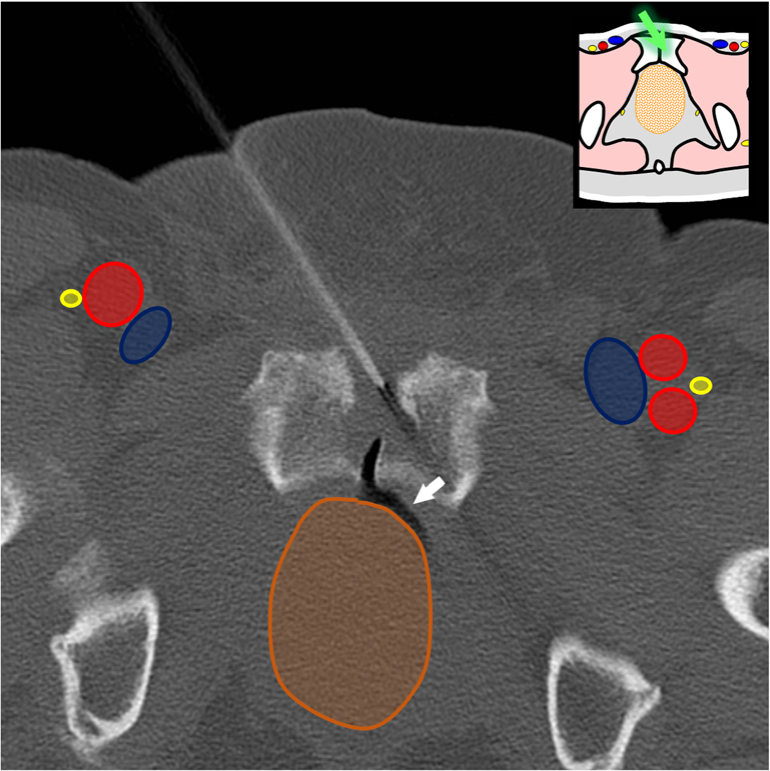
A 71-year-old man with destructive pubic symphysis arthropathy. An anterior approach was used to perform the biopsy with a 13-G bone needle. There is gas from the pubic symphysis diffusing to the Retzius space (*white arrow*)

- In the supine position, an anterior approach to the pubis can be used (Figs. 19 and 20).
- In the prone position, a posterior approach to the ischial tuberosity can be used.

## Technical considerations

For every pelvic bone procedure, great care should be taken to avoid pelvic viscera, vessels and nerves during the approach to the lesion. Depending on the kind of procedure, other potential risks must be considered, such as non-target ablation and extraosseous cement leakage.

### Biopsies

A well-planned approach for percutaneous bone biopsies is essential to obtain a representative sample of the lesion and to minimise procedural risks [4, 16, 24–26].

If a primary bone lesion is biopsied, gluteal muscles and rectus femoris should be avoided, as they are essential in a limb-sparing procedure and the needle tract would have to be resected [16, 26–28].

When a pelvic bone metastasis is favoured, intramuscular needle path is less determinant, albeit cases of tumour seeding along the needle path after core biopsy have been reported [25]. A direct access through gluteal muscles can be used provided that no major vessels or nerves lie in the needle path. However, reducing the length of the path in the soft tissues decreases the risk of bleeding. Moreover, iliac lesions tend to have a longer diameter in the wing axis and more material can be sampled by accessing the lesion along its greater axis.

The choice of the needle depends on the mineralisation of the lesion and the presence of a cortical breach [16]. A 14- to 16-G soft tissue cutting biopsy needle is the favoured choice whenever possible. A 10- to 16-G bone biopsy needle is indicated for dense lesions and when the cortex is intact. A coaxial technique is recommended because it allows keeping bone access to take several samples and occasionally perform percutaneous embolisation, and also protects the needle path from tumour dissemination [24, 25].

The number of samples required depends on the pathology department of each institution [3, 16, 24]. Usually two or three samples in formalin are enough [3, 24–26]. More samples may be needed when using smaller-gauge needles, mainly in paediatric patients [26]. If a lymphoma is suspected, a sample in saline serum should be sent to allow the realisation of flow cytometry [16]. Because infection is an occasional mimicker of bone tumours, systematically sending one or two samples for microbiological analysis is a good practice in uncertain cases [29].

### Percutaneous tumour ablation

Percutaneous tumour ablation can be curative or palliative [5–7, 30]. There are several techniques available with different indications: ethanol, laser, radiofrequency, microwave and cryoablation [5–7, 30].

**Fig. 21:**
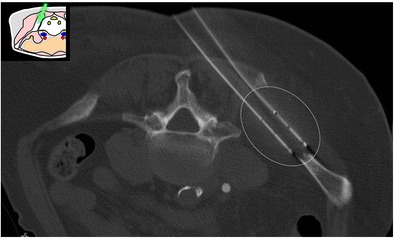
A 65-year-old woman with lung cancer and painful iliac metastasis. Axial CT shows the cryoablation probes inserted along the greater axis of the iliac wing and the hypodense ice ball covering the lesion (*dotted line*)

**Fig. 22:**
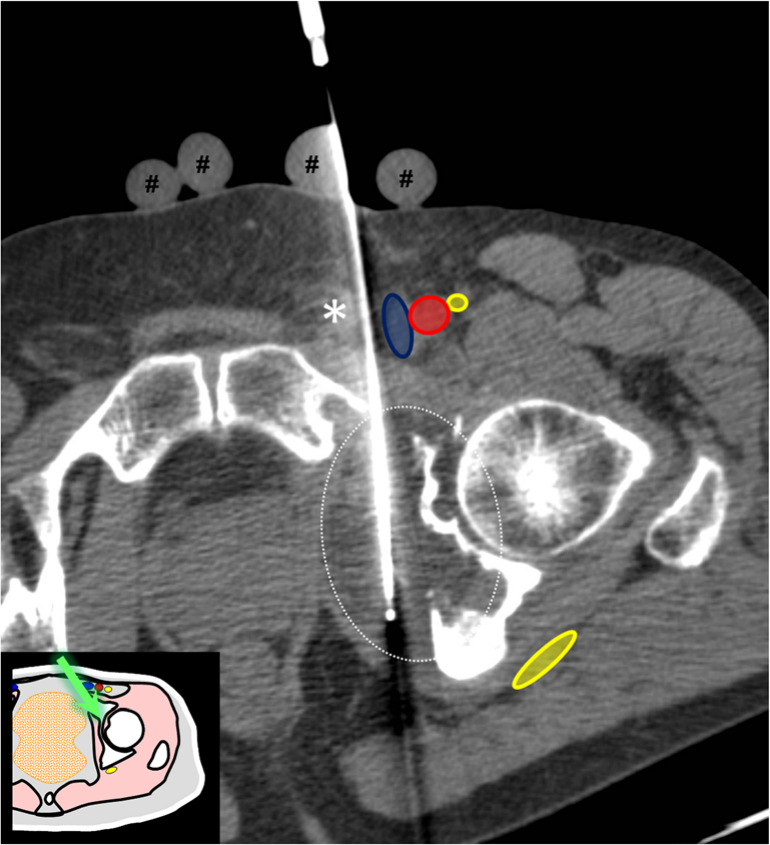
A 68-year-old man with bladder cancer presenting with painful acetabular metastasis. Axial CT shows one of the cryoablation probes inserted in the lesion with an approach between the spermatic cord (*asterisk*) and the femoral vein. The hypodense ice ball (*dotted line*) is seen. Note the glove fingers filled with warm saline over the skin to prevent thermal skin damage (#)

For a safe procedure, it is essential to carefully plan the approach in order to obtain a good coverage of the target lesion and avoid neighbouring critical structures [6, 7]. Frequently, insertion of several applicators is needed to obtain adequate lesion coverage (Fig. 21). MRI has the unique ability to monitor chemical and thermal variations in the treated area. With cryoablation, CT can also demonstrate the formation of an “ice ball” as a hypodensity in the treated area, although this is much more evident in the soft tissues than within the bone (Fig. 22) [5, 7, 16, 20, 30].

Different techniques have been described to prevent thermal damage to adjacent critical structures (mainly nerves and visceral structures), such as temperature monitoring to prevent overheating or overcooling, carbon dioxide gas or liquid dissection to increase the distance between the target area and critical structures and counteract temperature changes [30–32]. Covering the skin with sterile gloves filled with warm saline can also be helpful to prevent frost bites during cryoablation of superficial lesions (Fig. 22) [30].

### Cementoplasty

**Fig. 23:**
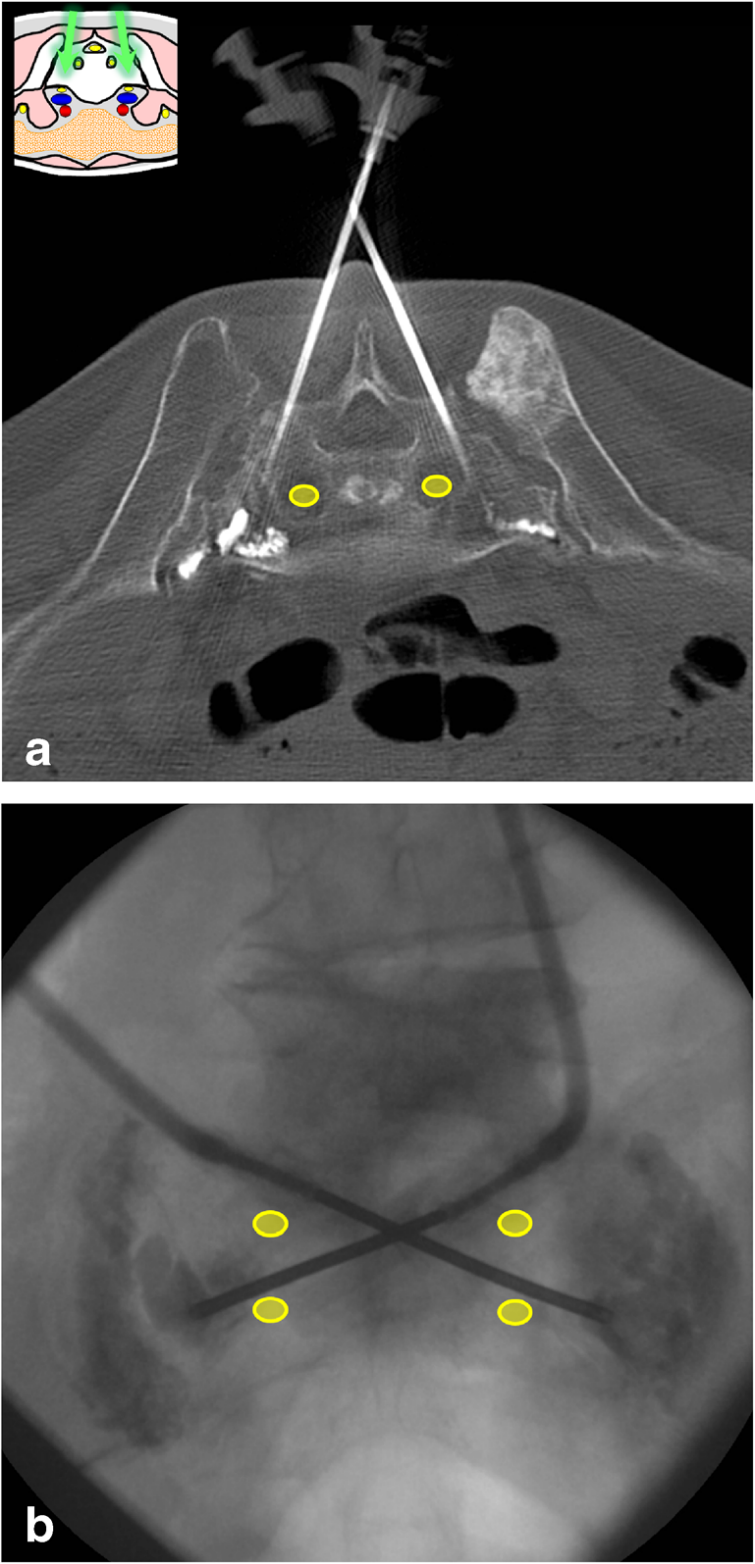
A 70-year-old woman with metastatic lung cancer and sacral insufficiency fractures. **a** Axial CT shows the 13-G bone needles inserted in the sacral wings and the beginning of the cement injection. **b** PA fluoroscopy view is essential to appreciate the real-time cement distribution and confirms the filling of the fracture lines on both sides. Special attention should be paid for sacral foramina during this procedure

Pelvic cementoplasty is used for pain management and bone reinforcement in certain cases of pelvic bone fractures and metastasis [8–16]. A well-planned approach will be determinant for optimising bone filling with acrylic surgical cement, while reducing the risk of extraosseous leakage (Figs. 16, 19 and 23). Treatment of extensive lesions may require the insertion of several needles to optimise bone filling [9]. After needle positioning, a pasty cement is injected under real-time imaging control in order to stop the injection when a satisfactory filling is obtained or a leakage is detected [10–13, 16]. Extraosseous cement leakage in the vicinity of neural structures (typically, sacral canal and foramina, and posterior aspect of the acetabulum) and into the hip joint should be avoided [9, 10, 12, 13]. Neural pain due to cement leakage next to a nerve can be treated with cortisone infiltrations around the affected nerve [10, 13]. Symptomatic leakage to the hip joint may uncommonly require surgical removal of the cement [9].

### Percutaneous screw fixation

**Fig. 24:**
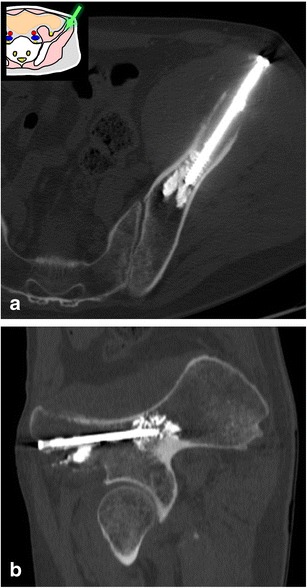
A 75-year-old man treated for a rectal adenocarcinoma presenting with a single painful metastasis in the left iliac bone and early pathological fracture. A cementoplasty combined with percutaneous osteosynthesis was performed along the greater axis of the iliac wing. **a** Axial CT and **b** sagittal oblique reconstruction show the screw and the cement in the iliac bone

Pelvic fixation with percutaneous screws can be used as a treatment for non-displaced fractures or to prevent fractures in patients with lytic metastases (Fig. 24) [14, 15]. The approach and the screw length can be safely planned with CT. In cases of osteolytic metastases, this technique is ideally combined with cementoplasty to allow a better fixation and support [15].

All these described techniques can be used alone or in combination to obtain better pain control and mechanical support [5, 15, 16]. When combining techniques, it is usually feasible to use the same coaxial access to the target area, in order to reduce the risk of complications along the approach and to reduce the procedural time (Figs. 11, 12, 19 and 24).

## Conclusions

In conclusion, pelvic bone procedures are safe to perform with an adequate knowledge of the anatomical landmarks. We described and illustrated multiple approaches to the most frequent targets at different levels of the pelvic bone. These approaches can be used for biopsies, percutaneous tumour ablation, cementoplasty, percutaneous osteosynthesis, or a combination of them. The principles of the different procedures and some practical and safety tips have been discussed as well.

## References

[1] Peh W (2006). CT-guided percutaneous biopsy of spinal lesions. Biomed Imaging Interv J.

[2] Liu PT, Valadez SD, Chivers FS, Roberts CC, Beauchamp CP (2007). Anatomically based guidelines for core needle biopsy of bone tumors: implications for limb-sparing surgery. Radiographics.

[3] Toomayan GA, Major NM (2011). Utility of CT-guided biopsy of suspicious skeletal lesions in patients with known primary malignancies. AJR Am J Roentgenol.

[4] Errani C, Traina F, Perna F, Calamelli C, Faldini C (2013). Current concepts in the biopsy of musculoskeletal tumors. ScientificWorldJournal.

[5] Moser T, Buy X, Goyault G, Tok C, Irani F, Gangi A (2008). Image-guided ablation of bone tumors: review of current techniques. J Radiol.

[6] Kurup AN, Callstrom MR (2010). Image-guided percutaneous ablation of bone and soft tissue tumors. Semin Intervent Radiol..

[7] Munk PL, Murphy KJ, Gangi A, Liu DM (2011). Fire and ice: percutaneous ablative therapies and cement injection in management of metastatic disease of the spine. Semin Musculoskelet Radiol.

[8] Cotten A, Duquesnoy B (1995). Percutaneous cementoplasty for malignant osteolysis of the acetabulum. Presse Med.

[9] Weill A, Kobaiter H, Chiras J (1998). Acetabulum malignancies: technique and impact on pain of percutaneous injection of acrylic surgical cement. Eur Radiol.

[10] Iannessi A, Amoretti N, Marcy PY, Sedat J (2012). Percutaneous cementoplasty for the treatment of extraspinal painful bone lesion, a prospective study. Diagn Interv Imaging..

[11] Sun G, Jin P, Liu XW, Li M, Li L (2014). Cementoplasty for managing painful bone metastases outside the spine. Eur Radiol.

[12] Masala S, Konda D, Massari F, Simonetti G (2006). Sacroplasty and iliac osteoplasty under combined CT and fluoroscopic guidance. Spine (Phila Pa 1976).

[13] Frey ME, Depalma MJ, Cifu DX, Bhagia SM, Carne W, Daitch JS (2008). Percutaneous sacroplasty for osteoporotic sacral insufficiency fractures: a prospective, multicenter, observational pilot study. Spine J.

[14] Deschamps F, de Baere T, Hakime A et al (2016) Percutaneous osteosynthesis in the pelvis in cancer patients. Eur Radiol 26:1631–1639 10.1007/s00330-015-3971-1 26318372

[15] Pusceddu C, Fancellu A, Ballicu N, Fele RM, Sotgia B, Melis L (2017). CT-guided percutaneous screw fixation plus cementoplasty in the treatment of painful bone metastases with fractures or a high risk of pathological fracture. Skelet Radiol.

[16] Hillen TJ, Baker JC, Jennings JW, Wessell DE (2013). Image-guided biopsy and treatment of musculoskeletal tumors. Semin Musculoskelet Radiol.

[17] Traina F, Errani C, Toscano A et al (2015) Current concepts in the biopsy of musculoskeletal tumors. J Bone Joint Surg Am 97:e7 25723000

[18] Gangi A, Kastler BA, Dietemann JL (1994). Percutaneous vertebroplasty guided by a combination of CT and fluoroscopy. AJNR Am J Neuroradiol.

[19] Hillen TJ, Talbert RJ, Friedman MV et al (2017) Biopsy of CT-occult bone lesions using anatomic landmarks for CT guidance. AJR Am J Roentgenol 209:214–221 10.2214/AJR.16.17468 28463540

[20] Guth SBX, Guermazi A, Gangi A, Gangi A, Guth S, Guermazi A (2009). Procedure basics and technique guidance. Imaging in Percutaneous musculoskeletal interventions.

[21] Braak SJ, van Strijen MJ, van Leersum M, van Es HW, van Heesewijk JP (2010). Real-time 3D fluoroscopy guidance during needle interventions: technique, accuracy, and feasibility. AJR Am J Roentgenol.

[22] Chehab MA, Brinjikji W, Copelan A, Venkatesan AM (2015). Navigational tools for interventional radiology and interventional oncology applications. Semin Intervent Radiol..

[23] Huegli RGT, Jacob AL, Messmer P, Gangi A, Guth S, Guermazi A (2009). Closed reduction and percutaneous fixation of pelvic fractures. Imaging in percutaneous musculoskeletal interventions.

[24] Le HB, Lee ST, Munk PL (2010). Image-guided musculoskeletal biopsies. Semin Intervent Radiol.

[25] Exner GU, Kurrer MO, Mamisch-Saupe N, Cannon SR (2017). The tactics and technique of musculoskeletal biopsy. EFORT Open Rev.

[26] Espinosa LA, Jamadar DA, Jacobson JA et al (2008) CT-guided biopsy of bone: a radiologist’s perspective. AJR Am J Roentgenol 190:W283–W289 10.2214/AJR.07.3138 18430813

[27] Anderson MW, Temple HT, Dussault RG, Kaplan PA (1999). Compartmental anatomy: relevance to staging and biopsy of musculoskeletal tumors. AJR Am J Roentgenol.

[28] Schwartz HS, Spengler DM (1997). Needle tract recurrences after closed biopsy for sarcoma: three cases and review of the literature. Ann Surg Oncol.

[29] Moser T, Ehlinger M, Chelli Bouaziz M, Fethi Ladeb M, Durckel J, Dosch JC (2012). Pitfalls in osteoarticular imaging: how to distinguish bone infection from tumour?. Diagn Interv Imaging.

[30] Filippiadis DK, Tutton S, Mazioti A, Kelekis A (2014). Percutaneous image-guided ablation of bone and soft tissue tumours: a review of available techniques and protective measures. Insights Imaging.

[31] Buy X, Tok CH, Szwarc D, Bierry G, Gangi A (2009). Thermal protection during percutaneous thermal ablation procedures: interest of carbon dioxide dissection and temperature monitoring. Cardiovasc Intervent Radiol.

[32] Tsoumakidou G, Buy X, Garnon J, Enescu J, Gangi A (2011). Percutaneous thermal ablation: how to protect the surrounding organs. Tech Vasc Interv Radiol.

